# Abdominopelvic Tuberculosis Secondary to a Nontuberculous *Mycobacterium* in an Immunocompetent Patient

**DOI:** 10.1155/2017/9016782

**Published:** 2017-11-13

**Authors:** Beng Kwang Ng, Kembang Aziah Yakob, Wendy Yin Ling Ng, Pei Shan Lim, Rahana Abd Rahman, Abdul Kadir Abdul Karim, Ani Amelia Zainuddin, Zaleha Abdullah Mahdy

**Affiliations:** ^1^Department of Obstetrics and Gynaecology, UKM Medical Centre, Kuala Lumpur, Malaysia; ^2^Department of Radiology, Hospital Kuala Lumpur, Kuala Lumpur, Malaysia

## Abstract

Tuberculosis (TB) remained as one of the top 10 causes of death worldwide despite an overall decline in its incidence rate globally. Extrapulmonary TB is uncommon and only accounts for 10–20% of overall TB disease burden. Abdominopelvic TB is the sixth most common location of extrapulmonary TB. The symptoms and signs are often insidious and nonspecific. Diagnosing abdominopelvic TB can be very challenging at times and poses great difficulties to the clinician. Infection with nontuberculous *Mycobacterium* (NTM) is even rarer especially in an immunocompetent patient. We report a case of NTM in abdominopelvic TB. A 37-year-old foreign worker, para 3, presented with a one-week history of suprapubic pain associated with fever. An assessment showed presence of a right adnexal mass. She was treated as tuboovarian abscess with intravenous antibiotics. Unfortunately, she did not respond. She underwent exploratory laparotomy. Intraoperatively, features of the mass were suggestive of a right mature cystic teratoma with presence of slough and cheesy materials all over the abdominal cavity as well as presence of ascites. Diagnosis of NTM was confirmed with PCR testing using the peritoneal fluid. This case was a diagnostic dilemma due to the nonspecific clinical presentation. Management of such rare infection was revisited.

## 1. Introduction

One of the targets in Sustainable Development Goals (SDGs) for 2030, adopted by the United Nations in 2015, is to end the TB endemic. The World Health Organization End TB Strategy calls for a reduction in TB deaths and incidence by 90% and 80%, respectively, by the year 2030 [1]. As reported by Global Tuberculosis Report 2016, there are an estimated 10.4 million of new cases and 1.4 million deaths due to TB worldwide in the year 2015. Although most prevalent as pulmonary disease, TB can affect any part of the body other than the lung, which is referred to as extrapulmonary TB. Most often, extrapulmonary TB is the result of haematogenous spread from primary pulmonary infection due to reactivation of a dormant disease. Abdominopelvic TB is one of the commonest locations of extrapulmonary TB. Predisposing factors include immunocompromised host, HIV infection, diabetes, malnutrition, alcohol, poverty, low socioeconomic status, and crowded environment with improper ventilation [2]. Infection with NTM is even rarer especially in an immunocompetent patient [3]. We report a case of NTM infection in a patient who was initially treated as tuboovarian abscess and eventually diagnosed to have abdominopelvic TB.

## 2. Case Report

**Figure 1:**
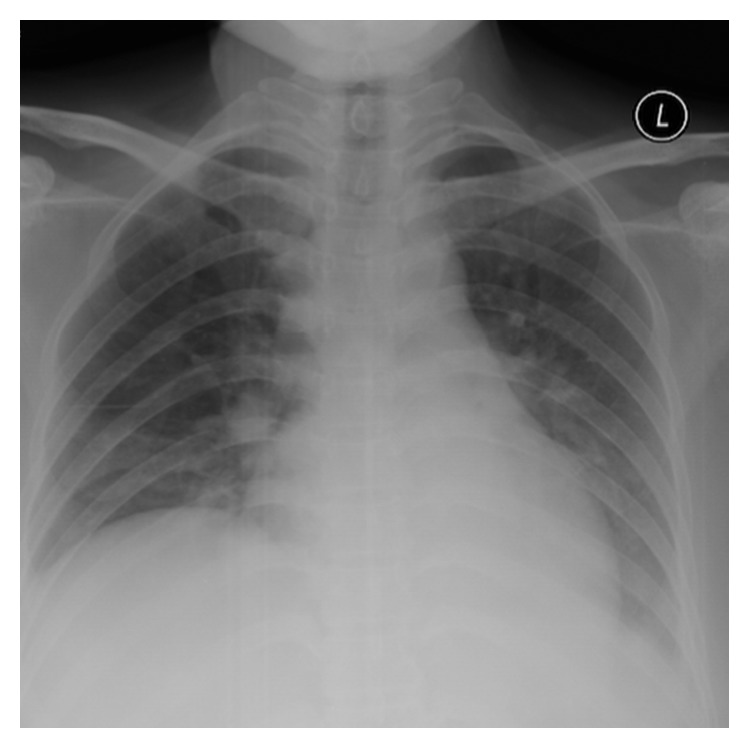
Chest radiograph revealed bilateral perihilar opacities which could represent enlarged hilar nodes. There was no evidence of fibrosis, pleural effusion, or infective changes.

**Figure 2:**
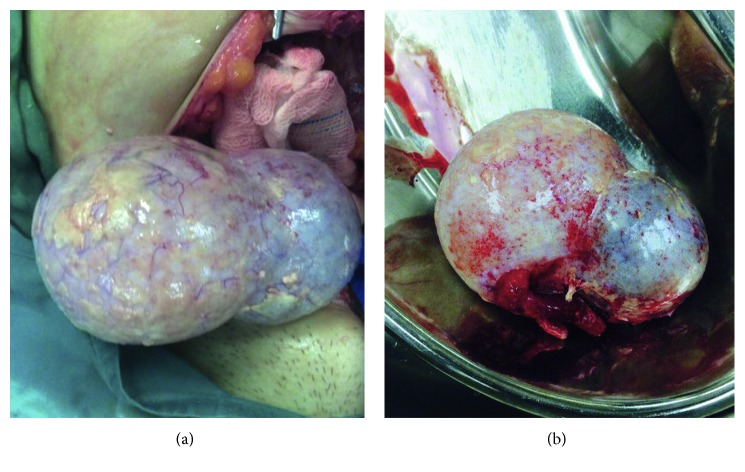
Laparotomy finding (a) and specimen (b) from right salpingo-ophorectomy with features suggestive of a mature cystic teratoma.

A 37-year-old foreign domestic worker with no history of major infection and 3 previous vaginal births presented with a one-week history of suprapubic pain associated with fever. The patient also experienced loss of appetite and loss of weight. On examination, she was tachycardic with high-grade fever of 38.9°C. Abdomen examination revealed tenderness over her suprapubic area; however, pelvic examination showed no abnormality. Her total white cell count was 5.9 × 10^9^/L, with predominantly lymphocytes (16.5 × 10^9^/L) and monocytes (2.6 × 10^9^/L). Her C-reactive protein (CRP) and erythrocyte sedimentation rate (ESR) were elevated at 29.9 mg/dl and 100 mm/Hr. Chest radiograph showed bilateral perihilar opacities which could represent enlarged hilar nodes. There was no evidence of fibrosis, pleural effusion, or infective changes (Figure 1). Ultrasound scan showed presence of right complex mass measured 12 × 6 × 6 cm with minimal free fluid at the pouch of Douglas. Cancer antigen 125 (CA 125) was elevated at 97 u/ml. Working diagnosis of tuboovarian abscess was made, and she was treated with a broad-spectrum intravenous antibiotic. Despite 48 hours of parenteral antibiotic, she did not improve clinically. After discussion, she opted for exploratory laparotomy. There were moderate amount of ascitic fluid with the presence of slough and cheesy materials covering the entire abdominal and pelvic cavities. However, there was no tubercle noted over the peritoneal surface. There was a right ovarian cyst measuring 12 × 6 cm and features suggestive of a mature cystic teratoma. Right salpingo-oophorectomy was performed (Figure 2). Peritoneal fluid cytology showed no malignant cells, and there was no acid-fast bacilli (AFB) seen on AFB staining. *Mycobacterium* culture did not yield any positive AFB even after 6 weeks. In view of high index of suspicion for TB, peritoneal fluid was also sent for polymerase chain reaction (semiauto TB PCR machine, Rotor-Gene Q®, Qiagen) testing, which was positive for NTM but negative for the *Mycobacterium tuberculosis* complex. Bronchoalveolar lavage was done as the patient was unable to produce sputum, which was negative for TB as well. Infective screening such as HIV, venereal disease research laboratory (VDRL), and hepatitis was nonreactive. Histopathological examination confirmed a right mature cystic teratoma. She remained well postoperatively and completed intravenous ceftriaxone and metronidazole for 10 days. She was initially planned for anti-TB (isoniazid, rifampicin, pyrazinamide, and streptomycin) following positive PCR testing for NTB. However, upon discharge, she was sent back to home town immediately by her employer. Hence, we have no further information about her progress.

## 3. Discussion

Extrapulmonary TB is uncommon. The incidence varies according to geographical location and study population. A review by Namani et al. at an infectious disease clinic in Prishtina reported that the incidence of extrapulmonary TB was 0.6% of their 3156 hospitalised patients. The reported incidence in eastern Sudan is almost similar at 0.9% [4]. Rajaram et al. reviewed 50 patients with chronic pelvic pain who underwent diagnostic laparoscopy. Gross findings of TB were seen in 26% of them and subsequently confirmed diagnosis of TB was 34% [5].

Abdominopelvic TB constitutes up to only 12–18% of extrapulmonary TB [6, 7]. It can occur in any organs such as gastrointestinal tract, viscera, peritoneum, or lymph nodes [8–10]. Most often, extrapulmonary TB is the result of haematogenous spread from primary pulmonary infection due to reactivation of a dormant disease. However, less than one-third of cases showed evidence of pulmonary TB, and only less than 20% of cases had past history or family history of pulmonary TB [11].

Liu et al. reviewed 28 cases of abdominopelvic TB, where up to 75% of patients presented with pelvic mass and ascites. Slightly less than half (46.4%) of the patients presented with fever and night sweat. There were only one-third of patients complaining of cough, while 10% presented with subfertility [12]. Another retrospective review of 20 cases by Xi et al. showed that the patient was younger at a mean age of 28.9 years. The main clinical manifestations were abdominal pain (45%) and abdominal distension (45%). Abdominal mass was only palpable in 7 cases (35%). Other presenting symptoms include weight loss, subfebrile fever, anorexia, diarrhoea, and frequent urination [11]. Thus, the clinical presentation of abdominopelvic TB was nonspecific and hence could mimic other pathologies such as ovarian malignancy, pelvic inflammatory disease, or tuboovarian abscess.

Almost all patients with abdominopelvic TB had elevated CA 125 level, which could be as high as 1440 IU/ml [10, 12]. Routine blood test showed elevated white cell count with predominantly neutrophils and lymphocytes in only 20% of cases. ESR and CRP were elevated in more than 85% of patients [10]. Abdominal ultrasound revealed a pelvic mass in 75–90% of patients with abdominopelvic TB, and 60–70% of patients had ascites [10, 12]. A normal chest radiograph could not exclude abdominopelvic TB as it was not always associated with pulmonary disease.

Our patient had elevated CA 125 level, and ultrasonography revealed a complex right adnexal mass with ascites. In view of fever with a complex ovarian mass, working diagnosis of tuboovarian abscess was made. As she did not respond clinically, exploratory laparotomy was performed. It had been reported in literature that pelvic TB can be misdiagnosed as ovarian malignancy or pelvic inflammatory disease (PID) based on the similarities in their clinical presentation and laboratory findings [6, 9–11]. Patients with PID or tuboovarian abscess usually have a history of frequent sexual coitus and acute onset with shorter duration of disease. Physical examination may reveal severe tenderness at the fornix due to peritoneal irritation. However, it tends to improve with antibiotic treatment [10]. In contrast, abdominopelvic TB frequently occurred in those suffering from chronic illness or immunocompromised. The onset is more insidious with longer duration of disease. Physical examination often showed no or mild tenderness at the fornix with mild peritoneal irritation sign. Patients often deteriorate even though given antibiotic treatment [10].

Diagnosis of abdominopelvic TB prior to surgical intervention is always challenging because the bacterial load is lower and sample collection can be difficult. Abdominal paracentesis is not helpful as it is usually negative for bacteriologic cultures and cytological studies. Ziehl-Neelsen staining can be negative in most cases [12]. However, presence of lymphocytes, mesothelial cells, and occasionally polymorphonuclear cells with no malignant cells may suggest abdominopelvic tuberculosis rather than malignancy [10]. The use of adenosine deaminase (ADA), which is a marker for T lymphocyte and macrophage activity arising from ascitic antigen of *Mycobacterium tuberculosis*, has been reported. Its sensitivity and specificity for the diagnosis of abdominopelvic TB are higher than 90% [10, 13]. It had been suggested that ADA testing should be done routinely for patients suspected to have TB peritonitis [10]. However, this test was not available in our centre. Newer assays such as QuantiFERON-TB Gold (QFT-G) have gained much interest and popularity in recent years. This is an IFN-gamma-release assay that measures the release of interferon after in vitro stimulation by *Mycobacterium* antigens [14]. As compared to the tuberculin skin test, QFT-G produces result immediately, and there is lack of cross-reaction with bacillus Calmette-Guérin and most of the NTB mycobacteria [14].

Therefore, diagnostic laparoscopy or laparotomy remained the best method in cases of diagnostic uncertainty and to obtain tissue sample for proper histopathological examination [12, 15]. In the review of 20 cases by Xi et al., laparotomy was performed in 85% of patients with presumptive diagnosis of ovarian cancer. However, all the patients were diagnosed with TB instead. Common intraoperative findings include widespread miliary nodules, adhesions, adnexal mass, and a caseous necrosis substance [12]. Rajaram et al. used the following laparoscopic criteria such as findings of tubercles, caseation, granulomas, beaded tubes, tuboovarian masses, fimbrial agglutination, and hydrosalpinx to suggest TB. Kappa measure of agreement between laparoscopic findings and peritoneal fluid PCR was 0.716, that is, substantial agreement [5].

Despite an array of diagnostic testing is available, diagnosis of abdominopelvic TB remained elusive. Frequently used conventional laboratory tests such as AFB smear, BACTEC with Lowenstein-Jensen culture, and HPE for *Mycobacteiurm tuberculosis* are flawed due to low sensitivity, time consumption, and delayed result. Molecular testing with PCR has been shown to be a useful adjunct to these conventional tests with better sensitivity [16, 17]. Our patient tested PCR positive for NTM in the peritoneal fluid. From literature, NTM species are mycobacterial species other than those classified to the *Mycobacterium tuberculosis complex* and *M. leprae*. Studies had showed that most of the NTMs were isolated from respiratory specimens although the overall incidence of pulmonary NTM infection was low [18]. Skin and soft tissue infections were the most common form of extrapulmonary NTM disease documented, accounting for 12% of all NTM diseases [19].

The mainstay of treatment for abdominopelvic TB is multiple drug regimens for a duration of 6–9 months. Combination of anti-TB treatments such as isoniazid, rifampicin, pyrazinamide, and ethambutol or streptomycin is commonly used [10, 11]. There was up to 40% of infected patients presented with subfertility later. Infection with mycobacteria causes direct damage to the fallopian tubes and thus their blockage [4]. The patient was counselled regarding this potential complication as she wished to conceive again.

In conclusion, clinical presentation of abdominopelvic TB is nonspecific. High index of suspicion is required to establish an accurate diagnosis of abdominopelvic TB so that prompt treatment could be initiated. Various laboratory and imaging tests should be performed in order to obtain a definitive diagnosis. This is crucial to avoid any misdiagnosis of this potentially fatal yet curable disease from other pathologies such as ovarian malignancy or tuboovarian abscess.
